# Location, Location, Location: Spatial Immune‐Stroma Crosstalk Drives Pathogenesis in Asthma

**DOI:** 10.1111/imr.70013

**Published:** 2025-02-24

**Authors:** Régis Joulia, Clare M. Lloyd

**Affiliations:** ^1^ National Heart and Lung Institute Imperial College London London UK

**Keywords:** airway wall, asthma, immune/stroma, inflammation, lung, spatial responses

## Abstract

Chronic lung diseases including asthma are characterized by an abnormal immune response and active tissue remodeling. These changes in the architecture of the tissue are a fundamental part of the pathology across the life course of patients suffering from asthma. Current treatments aim at dampening the immune system hyperactivation, but effective drugs targeting stromal or acellular structures are still lacking. This is mainly due to the lack of a detailed understanding of the composition of the large airways and the cellular interactions taking place in this niche. We and others have revealed multiple aspects of the spatial architecture of the airway wall in response to airborne insults. In this review, we discuss four elements that we believe should be the focus of future asthma research across the life course, to increase understanding and improve therapies: (i) specialized lung niches, (ii) the 3D architecture of the epithelium, (iii) the extracellular matrix, and (iv) the vasculature. These components comprise the main stromal structures at the airway wall, each playing a key role in the development of asthma and directing the immune response. We summarize promising future directions that will enhance lung research, ultimately benefiting patients with asthma.

## 1 Introduction

The respiratory tract and all its components are the result of centuries of evolution and adaptation to an environment that has shaped the cells and anatomical structure. Indeed, this complex organ system is divided into three areas: the upper airways (i.e., nasal cavity, pharynx, and larynx), the lower airways (i.e., trachea, bronchi, and bronchioles), and finally, the parenchyma or respiratory zone comprised of alveoli [1, 2, 3, 4]. These specialized areas each have defined physiological functions: the upper airways are responsible for filtering, warming, and humidifying the air; the lower airways control gas exchange through breathing movements, while the respiratory zone facilitates gas exchange.

Maintaining lung respiratory health in the modern world presents a distinct and ever‐increasing challenge. The combination of climate change, increased pollution, emergent infectious diseases, and obesity has resulted in a hostile inhaled environment to which the lungs are constantly exposed [5, 6]. Our pulmonary immune system has evolved for thousands of years and has developed elaborate strategies to combat respiratory pathogens and facilitate tolerance to innocuous inhaled particles [7]. Innate and adaptive immune cells are the main effector arms of these strategies, and we and others have contributed to the elucidation of the distinct roles these different cell populations exert during lung‐protective and pathologic responses [2, 8, 9, 10, 11, 12].

In this context, asthma is a multifactorial inflammatory disease, predominantly characterized by its chronicity and impact on the lower airways [10, 12, 13, 14]. Environmental mediators such as allergens (e.g., house dust mites [HDM], pollen, or animal dander) and pollutants are known triggering agents for the clinical features of asthma that include coughing, wheezing, and airway hyper‐responsiveness (AHR) [15]. Research over the last decades has highlighted the role of Type 2 immunity in driving some of the pathophysiologic symptoms of asthma [16], and this has led to a series of biologic agents designed to dampen Type 2 immune cells or their secreted cytokines [17]. However, the fact that Type 2 therapies do not work in all patients, coupled with the advent of more detailed clinical phenotyping, indicates that asthma is a heterologous disease, with symptoms and prognosis differing in each patient. Due to the lack of curative treatments and the fact that asthma generally begins in childhood, many patients develop a chronic progressive disorder that culminates in poor lung function later in life, [9]. Asthma has reached epidemic proportions across the globe, and we are now at a turning point in lung research where we need to better appreciate the function of both the canonical immune cells present in the lungs, but also the impact of the local structural cells within the lung. We need to understand their influence on the immune response and assess the interactions between these structural cells and both resident and infiltrating immune cells within their local ecosystem. New technologies, improved analysis pipelines, and better in vitro and in vivo approaches developed in recent years will soon contribute to a new generation of knowledge and improved understanding of asthma and other respiratory disorders.

Here, we summarize four elements of the lung response to external stimuli specifically at the bronchial wall, as this is where the highest accumulation of immune cells and prominent tissue remodeling occurs in asthma, which we and others have explored in a spatiotemporal context.

## 2 Importance of Specialized Spatial Regions of the Lungs

**FIGURE 1:**
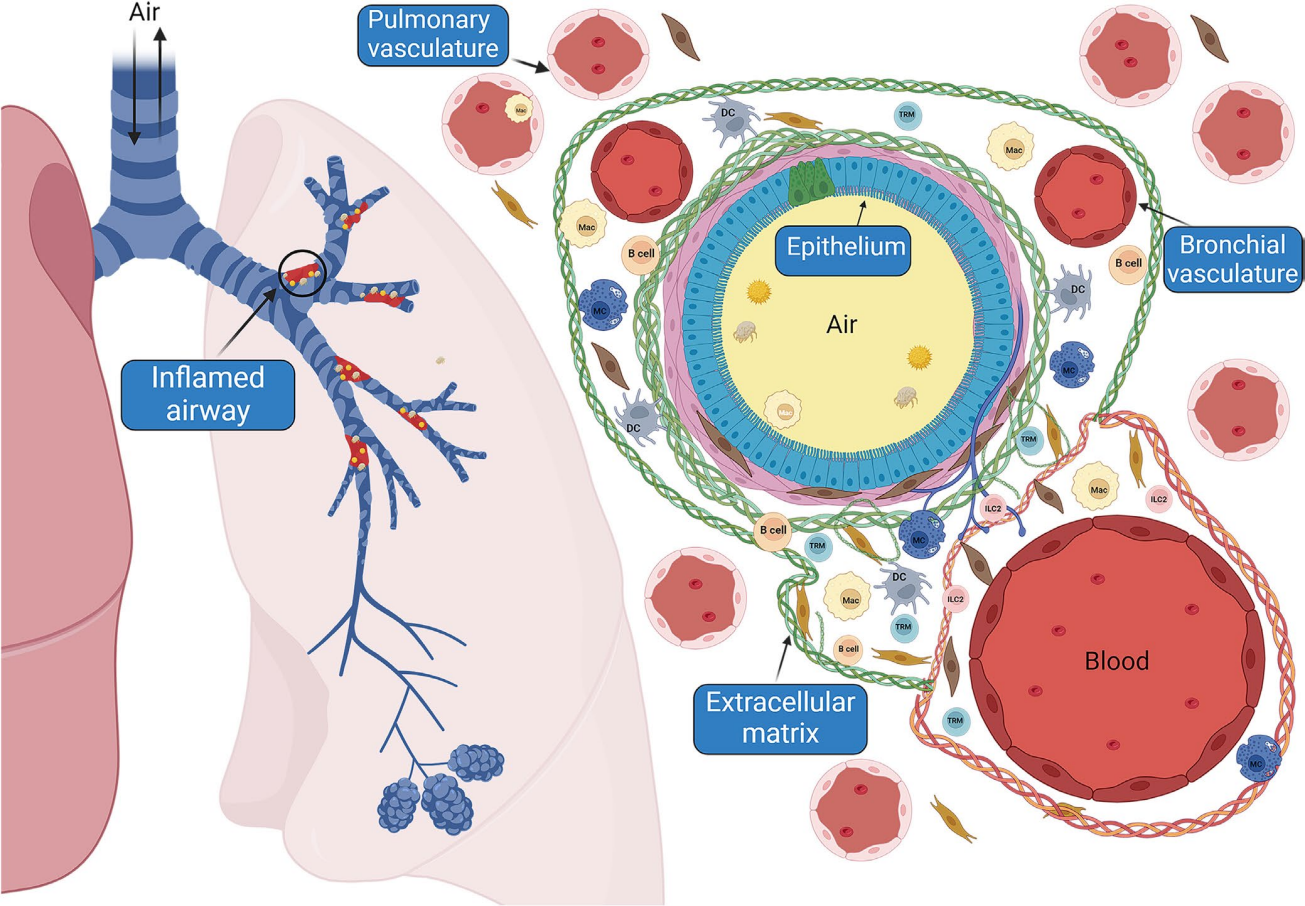
Organization of large airways. The respiratory tree is a stratified structure with a high level of cellular diversity organized spatially with asthma known to impact specific regions within the airways.

One key element missing from our understanding of asthma is the spatial location of tissue damage (Figure 1). The lungs are special in that they are relatively accessible because they are exposed directly to the external environment. Sampling of the lower airways is possible via a technique known as bronchoscopy, where a flexible tube is passed through the nose or mouth so that saline can be instilled into the airway and retrieved to gain a sample of the cells within the airway lumen (bronchioalveolar lavage, BAL). In concert, the walls can be “brushed” to obtain samples of cells lining the airways (Epithelial brushes), or a small sample of the airway wall tissue (biopsy) can be collected. Together, these samples allow phenotyping of a range of cells within the airways and from the airway wall that can provide a snapshot of the disease. These different samples will all yield different information, reflecting pathology according to location. For example, our study analyzing the immune‐proteomic landscape post–COVID‐19 infection compared cells from bronchoalveolar lavage (BAL) and blood samples from patients. We observed that while the blood signature returned to that of control patients after 3–6 months, the profile of the BAL remained distinct from control samples. In addition, the presence of immune cells such as neutrophils and B cells in the BAL correlated better with CT abnormalities in these patients [18].

Moreover, given their various physicochemical properties, inhaled environmental triggers are not uniformly distributed throughout the lung. We know from studying mouse models and human data that not all airways along the respiratory tree show signs of inflammation and remodeling following allergen exposure. This is inherent to the intricate structure of the lungs and complex airflow movements that will determine where particles are deposited [19, 20]. Hyperpolarized gas MRI studies have highlighted marked heterogeneity of ventilation in patients with asthma [21, 22]. The increase in airflow obstruction in specific areas has been shown to correlate to some extent with disease severity, as measured by lung function parameters such as FEV1 [23, 24]. The impact of cardiac function on the deposition of particles and airflow is still an active and complex area of investigation, where age and sex differences play an important role [25]. Future research to advance imaging tools and prediction models will help improve the treatment of specific regions within the lungs, potentially offering patients a more targeted, personalized medicine approach. One could envisage that this precision medicine tactic, similar to that applied in cancer treatment, whereby only a specific area of inflamed and remodeled bronchial wall could be targeted for treatment and thus improve the efficiency of therapy.

The revolution in spatial biology tools and improved access to patient samples has been transformative in our quest to better understand how the spatial location dictates the function of immune cells in situ. Most of our current knowledge of the lungs is based on single‐cell suspensions, either from BAL or from enzymatically dissociated tissue. The methods to achieve such dissociation result in a bias for those cell types accurately isolated and thus may not represent the true distribution of cells within the tissue. An example of this that is particularly pertinent to asthma is the difficulty in obtaining transcriptomic information from eosinophils in tissues by common single‐cell transcriptomic methods [26]. Furthermore, the generation of a single‐cell suspension removes the spatial context from where these cells originated. Recent works using human tissue samples clearly demonstrate the importance of spatial niches in the regulation of B cells, T cells, and γδ T cells during lung development and for immune memory [27, 28, 29]. Indeed, using pediatric organ donors, it was shown that B cells and T cells accumulated next to the large airways, forming clusters of cells during the first 3 years of life. These B cells secreted antibodies against respiratory pathogens not found in the plasma. This discovery highlights the need to investigate cells within their local ecosystem and the importance of maintaining spatial data across spatial ‘omic platforms’ [29].

Mechanistically, the interactions between immune cells and stromal cells have been investigated in detail using mouse reporter strategies and quantitative imaging. We and others have shown that the activation of immune cells in specialized regions of the lungs, such as the adventitial cuffs, have specific modes of immune regulation, including the production of interleukin 33 (IL‐33) [30], chemokines, and extracellular matrix (ECM) deposition [31]. Indeed, our data have shown that dysregulation of this niche can initiate airway remodeling via hyperactivation of mast cells and induce vascular damage [32]. These works and many others clearly show the importance of investigating disease‐specific regions of damage and that extrapolating data from blood samples may not be sufficient to discern meaningful conclusions in the context of asthma [33].

Based on these findings, moving forward, studies need to include specific details regarding the tissue type and location utilized in each experiment in order for accurate conclusions and comparisons to be drawn. This is equally important for both mouse and human samples in the era of omics analyses to avoid mixing datasets from different lung regions.

## 3 Zooming‐In on the Cells Forming the Bronchial Epithelium

The lung epithelium comprises a community of cells with highly specialized functions that can adapt with age and the cumulative exposures they encounter across the life course. It has been clearly established that the epithelium of the bronchi is injured during all forms of asthma, and the mechanisms driving tissue damage, such as disruption of tight junctions, E‐cadherin [34], and impaired barrier function [35], have been extensively reviewed [15].

Our knowledge of the cells that form the bronchial epithelium has radically changed from what we perceived a decade ago. Uncovering the complexity of this multilayered barrier of cells using modern tools led to the discovery of an unappreciated diversity of cells, whose functions remain to be fully elucidated. Large consortiums such as the lung cell atlas project (https://www.lungcellatlas.org/) [3, 26, 36, 37] have provided a formidable resource for the scientific community to explore and compare their datasets. Here, we discuss the current understanding of these epithelial barrier cells, not by designated cell types but according to their spatial orientation/polarization, how the latter dictates their function, and how this is altered during asthma.

The predominant epithelial cells of the bronchial epithelium that are the first to encounter inhaled air are the ciliated cells and secretory cells. Secretory cells secrete mucus that traps inhaled pathogens, pollution, and allergens, while ciliated cells possess cilia on their apical surfaces. These cells work together to move these trapped particles up the respiratory tree and out of the lungs [38, 39]. Our knowledge of ciliated cells beyond this basic function remains limited. It is unclear if these highly differentiated cells have additional functions in sensing changes in their microenvironment that could be crucial in the context of asthma and other respiratory disorders. Indeed, in an air/liquid interface (ALI) in vitro model, data have shown that viruses such as SARS‐CoV‐2 target ciliated cells and disrupt cilia beating [40]. This is also true for other pathogens, such as influenza, respiratory syncytial virus (RSV) or rhinovirus; bacteria, and fungus; all key players in asthma pathophysiology [41]. How different epithelial subtypes sense changes in the inhaled environment and detect potential threats that would influence barrier function is becoming an increasing area of interest. It is perhaps particularly critical in the context of asthma, especially in children. Although symptoms of most lung diseases are exacerbated during infections, children with viral infections are particularly affected, often leading to hospitalization [9, 42, 43]. Understanding how ciliated cells sense and respond to viral infection represents an intense area of investigation. One important aspect of the biology of these barrier cells is their organization. They are not present as a single layer but appear as multiple layers. Indeed, only the air‐exposed cell layer has beating cilia, and this is highly regulated genetically [37]. We know less about the immune functions of the other deeper layers. One could imagine that the most superficial layer would possess an increased capacity to detect microbiome/threats via an increased expression of pattern recognition receptors (PRRs). It is possible that the spatial organization of ciliated cells may drive the accumulation of pathogens in specific areas, and specific ciliated cells may then be equipped to detect these threats. However, the mechanisms by which this information is relayed to the deeper layers remain enigmatic. The remodeling of the airway wall in the context of asthma may lead to a disruption of the normal cellular organization and thus lead to disturbed cellular communications between the apical and more basal layers of the ciliated epithelial cells. In addition, the availability of oxygen and nutrients may be impacted by this spatial disorganization and remodeling; yet the impact of hypoxia specifically in ciliated cells is relatively unknown. Overall, the disorganization of this cellular ecosystem may play a critical role during the early development of asthma. Only by assessing these changes in situ will we be able to answer pertinent questions, and one of the most promising ways to do this is through the use of novel transcriptomic and spatial tools.

Secretory cells are positioned alongside ciliated cells on the luminal side of the epithelium. In addition to secreting mucus to trap pathogens, these cells also secrete antimicrobial molecules. This family of epithelial cells has expanded dramatically during the last decade and now includes goblet cells, club cells, tuft cells, pulmonary endocrine cells (PNECs), and serous cells [3]. Within the secretory epithelial cell family, the discrepancy between human and mouse lungs is more evident, with a high level of diversity and quantity of these cells observed in human airways, whereas mouse large airways show fewer secretory cells at steady state [44]. Goblet cell hyperplasia and mucous plugging have always been a classical feature of asthma, and the functions of this mucus during a lung immune response remain elusive, despite widespread evidence of its involvement in cases of fatal asthma [45, 46, 47]. It is beyond the scope of the review to discuss the role of mucus cells in detail, and thus we refer to excellent works for further reading [11]. It is important to note that no current therapy has proven to be effective in reducing mucus production in lung disease. However, exciting new data determining the biochemical and biophysical alterations of mucus that lead to mucus plugging in severe and fatal asthma may be a step in the right direction [11]. Identification of crystals within mucus that contain the protein galectin‐10 (Gal10) acts as an adjuvant for Type 2 immunity, thereby increasing inflammation, but antibodies specific for Gal10 dissolved these crystals and ameliorated disease symptoms in a humanized mouse model of asthma [48]. This may represent a novel therapeutic for severe asthma.

Recent spatial transcriptomic data have identified more complexity regarding secretory cells, with distinct gene signatures for subsets of spatially distributed secretory cells that may represent a spatial niche important for regulating inflammation [3]. Airway submucosal glands are very abundant in the human bronchial space, and following the production of C‐C motif chemokine ligand 28 (CCL28), APRIL, and interleukin 6 (IL‐6), B cells and plasma cells are recruited to these sites [3]. How this niche, characterized by steady state, adapts to chronic inflammation remains unknown. Additional work using tissue samples from patients with asthma will confirm the importance of this specialised area. Furthermore, using mouse models alone to determine the fine mechanisms of this niche will not be enough, as they do not mimic the complexity observed in human large airways.

Finally, the deepest layer of the airway epithelium is comprised of basal cells. Basal cells are the progenitors or stem cells of the airway epithelium, believed to be relatively undifferentiated and thus capable of repopulating the upper layers of the epithelial wall. More recently, basal cells have been shown to migrate to sites of injury [49]. Indeed, in the context of idiopathic pulmonary fibrosis (IPF) our recent data showed that KRT5^+^ basal cells not only migrate through the fibrotic lung but that their behavior and function were impacted by the ECM microenvironment. Notably, secreted protein acidic and cysteine rich (SPARC), a matricellular protein known to bind ECM components and coordinate ECM remodeling, was able to restrict KRT5^+^ basal cell migration [50]. The clinical manifestation of IPF occurs much later than the pathological changes that occur within the lungs, with patients being diagnosed at later stages of the disease. As such, the fibrotic changes and specifically the ECM remodeling are more advanced and widespread throughout the lung compared to that seen in tissue from asthmatic lungs. However, it is likely that common mechanisms, particularly during the early stages of disease, exist across all fibrotic lung diseases.

Basal cells are in a unique position at the interface between the ciliated cells and the inner environment, making them distinctively able to regulate the downstream immune process. One of the key mechanisms of immune regulation comes from the swift expression of alarmins such as IL‐33, thymic stromal lymphopoietin (TSLP), or interleukin 25 (IL‐25) following stimulation/activation. Lung basal cells are one of the main sources of alarmins, together with endothelial cells at steady state and during asthma [51]. A large body of research over the past decade into the role of these cytokines in asthma has led to the development of blocking antibodies to reduce the Th2 response. Indeed, targeting alarmins during asthma has shown great potential, with a range of novel biologics currently in clinical trials for asthma, such as itepekimab targeting IL‐33 [52], astegolimab targeting IL‐33 receptor ST2 [53], or tezepelumab targeting TSLP [54, 55]. In addition to the above, other alarmins are now being investigated, including a tumor necrosis factor family cytokine, TL1A, expressed in basal cells that has been shown to promote ILC2 cytokine expression in cooperation with IL‐33 [56].

The detailed functions of these alarmins have been extensively reviewed elsewhere [57]. Here, we discuss the importance of the spatial location of release and activation of these cytokines. While the cellular pattern of alarmin expression in mice is different from that observed in humans, it is interesting to note that alarmin expression is generally restricted to specific cell types in precise locations in both species. One would have thought that a vast repertoire of cells able to express alarmins would promote a swifter response by the immune system. However, this does not seem to be true for alarmins released by the epithelium. One possible explanation is the creation of “hot spots” of immune cell recruitment in the subepithelial space that could maintain immunological information locally and create specific tissue “training” for leukocytes [29, 58]. This concept is reminiscent of a chemokine gradient leading to a site of maximum production. Epithelia at the bronchial wall could use similar strategies to direct leukocytes to a specific site of infection or damage. We found that the location of neutrophils within the airway wall had implications for clinical phenotype. Those children with severe therapy‐resistant asthma who showed increased neutrophils in close proximity to the airway epithelial layer showed better lung function and improved symptom control compared to those who did not exhibit neutrophils in this specific location [59]. Molecular mechanisms driving these interactions between stroma and immune cells are still to be discovered, but alarmins and chemokines likely play an essential role. Understanding how these hubs, or cellular communities, are disrupted during asthma could be one of the earliest steps in the inflammatory cascade leading to the establishment of chronic inflammation. Therefore, it is critical to continue to investigate how the epithelium changes with progression down the respiratory tree but also temporally during development, and how chronic inflammation influences these changes. Finally, fascinating data have recently emerged documenting the role of the developing pulmonary immune system and its contribution to shaping epithelial cell fate [36]. IL‐1β released from leukocytes was shown to promote epithelial cell differentiation in vitro, and IL‐1β^+^ cells were found in close vicinity to the developing epithelium [36].

**FIGURE 2:**
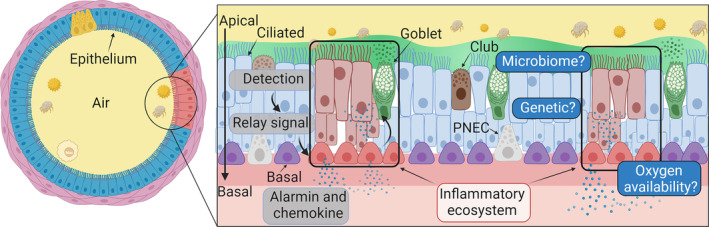
Spatial organization of the large airway epithelium. Epithelial structure dictates the functions of cells but how subpopulations of cells are triggered in asthma and how signals are organized spatially remain to be explored.

Other cells present in the lung bronchial epithelium include tuft cells, ionocytes, hillock cells, and microfold cells whose functions have been extensively reviewed elsewhere [60]. Information as to the specific location and interactions of these cells during health and asthma in humans remains relatively sparse and will require further exploration to determine the nature of these interactions and assess their importance (Figure 2).

In summary, despite our extensive and still growing knowledge of the epithelial cells at the bronchial wall, a better understanding of all subtypes within their specific microenvironment and how they regulate immune cell functions across age and disease is required to provide better treatments for asthma.

## 4 The Extracellular Matrix, a Constantly Moving Scaffold

To better understand the function of the lungs during health and disease, it is necessary to consider not only the cellular landscape but also the acellular structures that form the local milieu. The ECM is an essential component of every organism whose function greatly extends beyond maintaining the shape of organs [61, 62]. Lungs are enriched in ECM components such as collagens, fibronectin, elastic fibers, and hyaluronan. The composition of this ECM evolves as the structure of the lung changes throughout the respiratory tree, that is, trachea, bronchial, or parenchyma, facilitating the essential functions of the lungs. Regulation of the lung ECM in both mice and humans is dynamic, with constant remodeling and modification occurring following production and degradation via enzymatic activity [63]. Disruption of this process, resulting in increased deposition of ECM during asthma, is considered a canonical feature of the disease. Indeed, epithelial basement membrane and collagens (i.e., collagens I, III, and IV) are increased in the bronchial regions [64, 65]. Furthermore, the balance of matrix metalloproteinase (MMP) regulating ECM is also disrupted [66].

### 4.1 ECM Spatial Distribution and Interaction With Immune Cells

The concept of spatial organization is again key to understanding the role the ECM plays at the airway wall. The nature of lung ECM reflects the complexity of the respiratory tree and its specific niches, and investigation into localized ECM disruption is critical for a better comprehension of diseases. Certainly, how these ECM changes are disrupting immune cell activity in asthma remains relatively unknown. We and others have shown that immune cells use the ECM scaffold to migrate through the lungs. Using an IL‐33 mediated lung inflammation model, we showed that innate lymphoid cell 2 (ILC2) was actively recruited to specific locations of the airway wall, such as the adventitial cuff, at the intersection between the large blood vessels and the large airways. ILC2s were most prominent in areas with high levels of ECM. Using a gray‐level co‐occurrence matrix (GLCM) technique [67], we showed that the geometry of ECM fibers was modified compared to healthy airways. We demonstrated that the motility of ILC2 was dependent on chemokines (i.e., CCL8) distributed on ECM components. Individual collagens promoted changes in ILC2 shape and motility when tested in vitro. Using in vivo imaging techniques, ILC2s were shown to be highly dynamic, moving in peribronchial and perivascular spaces that were enriched for ECM. Collagen I specifically was shown to change ILC2 morphology via remodeling of their actin cytoskeleton. This dynamic motility was vital for regulating eosinophilic inflammation. Moreover, the use of a drug to disrupt collagen fiber assembly had a profound effect on ILC2 migration in vivo, with cells being cleared from the tissue. This reduction in ILC2 in the lungs was shown to ameliorate subsequent eosinophilic inflammation. Thus, in vivo, collagen‐I fibers may support a more polarized ILC2 morphology, thereby increasing ILC2 traction, reducing ILC2 speed, and allowing for longer dwell times in specific sites in the lung that enhance inflammation [31]. These data underscore the importance of the tissue matrix influencing local cellular functions and therefore impacting the magnitude and nature of tissue inflammation.

This study opens different avenues of research for lung biology and asthma. Given that changes in ECM are not only characterized by a change in the quantity of matrix components, as well as a balance in different types of matrix proteins, but the impact on immune cell functions should be determined after different inhaled stimuli. In addition, since the dynamics of ECM change with age and development, we need to understand if these changes are different across the life course and if this impacts the activity of other immune cells. To underscore the potential importance of this, we have shown that an increased reticular basement membrane formed by collagens and laminins is one of the earliest events present in childhood asthma and correlates with the severity of the disease [68, 69].

We and others have shown the ability of chemokines to bind ECM components, including glycosaminoglycans [31, 70]. Changes in the amount and geometry of the ECM may greatly influence the availability of these mediators to stimulation and therefore could alter the regulation of immune cell functions locally.

### 4.2 Localization of ECM‐Producing Cells

Cells capable of secreting ECM components have become a key focus of investigation. The ultimate aim of this body of research is to understand disease pathogenesis but also to begin to develop methods to manipulate the production of matrix proteins. We and others have shown that human fibroblasts produce large quantities of ECM. Recent analysis of IPF patient‐derived fibroblasts using specialized proteomics designed to focus on the “matrisome” revealed a distinct ECM signature in patient fibroblasts that was marked by increased SPARC and SERPINF‐1 expression [50]. Recent studies using transgenic reporter mice have also identified the primary ECM‐producing cells in the lungs [71]. *Col1a1*‐EGFP mice were used to highlight subpopulations of fibroblasts as the main source of collagen I, and exposure to bleomycin (as a model of IPF) dysregulated the expression of collagen by these cells [71]. These collagen I–secreting fibroblasts were genetically distinct and showed specialized spatial distribution in alveolar, adventitial, and peribronchial regions of the lungs. These data demonstrate the importance of combining in‐depth molecular phenotyping (scRNA‐seq) together with advances in imaging to fully appreciate the functions of ECM‐secreting cells within tissues and to understand their functional locations in health and disease. Improvements in clearing methods and whole‐organ imaging are making it possible to fully determine the localization and functions of these cells at the bronchial wall and investigate whether these cells are present along the whole respiratory tree or at specific points. The importance of these collagen‐expressing cells during asthma and how respiratory allergens are impacting their functions remain to be determined.

### 4.3 Importance of Localized Mechanical Forces at the Airway Wall

Another critical aspect of the biology of the ECM is its capacity to respond to mechanical stretch. Lung breathing movements constantly expose cells to dramatic variation in expansion and contraction, but how these forces impact the cellular ecosystem locally remains poorly understood [72]. Investigating the role of mechanical stretching is extremely complex in vivo and in vitro since the extent of these forces will vary considerably along the respiratory tree and will depend on the presence of inflammatory infiltrates, as well as any changes in matrix deposition. It was recently shown that pulmonary fibrosis could be driven by elevated mechanical tension in alveolar Type 2 cells (AT2) inducing a TGF‐β signaling loop leading to impaired alveolar regeneration [73]. While not directly relevant for asthma and the bronchial wall, this study clearly establishes that mechanical forces play a critical role in lung biology and repair following injury. Given that TGF‐β signaling has been shown to play a prominent role in the development of asthma inflammation, any local change in mechanical force in the bronchial wall may trigger a similar pathway.

The sensing of the physical movements in the lungs is regulated by mechanosensitive ion channels such as the transient receptor potential (TRP) superfamily and Piezo channels (i.e., Piezo 1 and Piezo 2), both of which are widely expressed throughout the lungs [74, 75]. Recently, an elegant study showed that Piezo1 is induced in pulmonary ILC2s upon activation. Using an IL‐33‐induced lung inflammation model, they showed that Piezo1 restrained ILC2 activity by reducing cytokine production, resulting in decreased airway hyperactivity [76]. This study suggests that modulation of mechanical forces during asthma may be a way to control immune cell activation. Again, gaining spatial information will be key to further understanding where these sensors are acting along the respiratory tree.

In the context of asthma, another recent exciting study characterized how bronchoconstriction observed during severe asthma leads to epithelial cell extrusion. Using ex vivo lung slices exposed to bronchoconstrictors such as methacholine, the authors elegantly showed that epithelial cells were expelled from the large airways into the lumen. Blocking mechanical pathways using inhibitors such as gadolinium (i.e., Piezo 1 inhibitor) reduced inflammation and mucous secretion [77]. This study indicates the fundamental concept that mechanical stretch is an important factor to consider in the lungs during inflammation and could be a method to regulate epithelial crowding during chronic disease.

**FIGURE 3:**
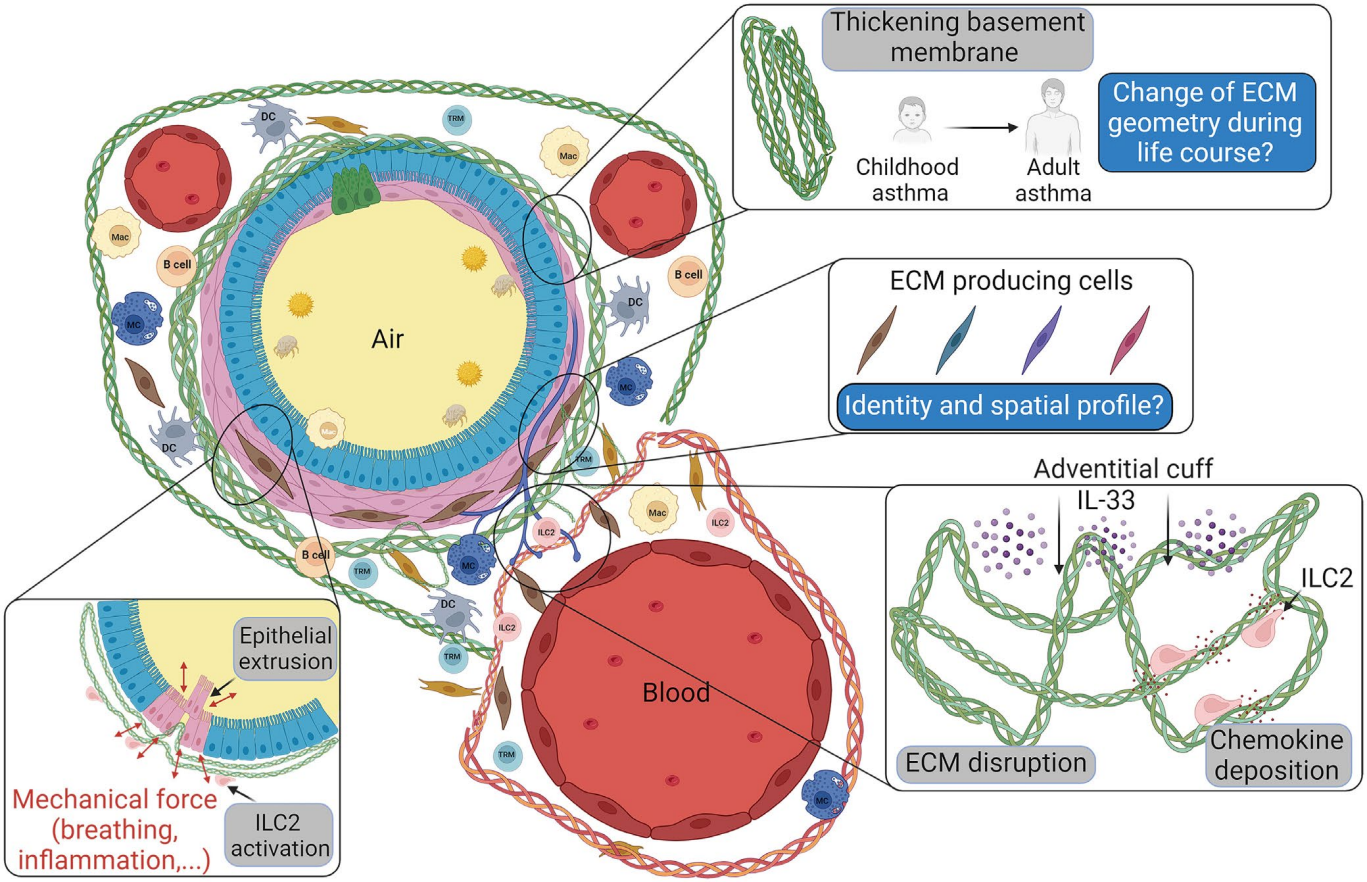
ECM in the large airways. ECM plays a key role in the airway wall beyond its scaffolding function. How asthma is disrupting ECM functions and the ECM‐producing cells remains to be determined.

Our understanding of the role of the ECM in lung disease is expanding, but many questions remain. Going forward, it will be critical to fully characterize the spatial location of different ECM components and identify their direct cellular interactors, particularly during asthma (Figure 3). One key characteristic of the lung ECM is that it is highly autofluorescent, making immunohistochemistry challenging; however, this characteristic does have an advantage in that it allows collagens and potentially other key components (e.g., elastin) to be directly imaged without the need for staining. One exciting new avenue of research to explore in the context of asthma is to measure ECM changes using noninvasive tools such as MRI or OCT scans [78].

## 5 Vasculature at the Airway Wall: Is Asthma Really an “Angiogenic” Lung Disease?

**FIGURE 4:**
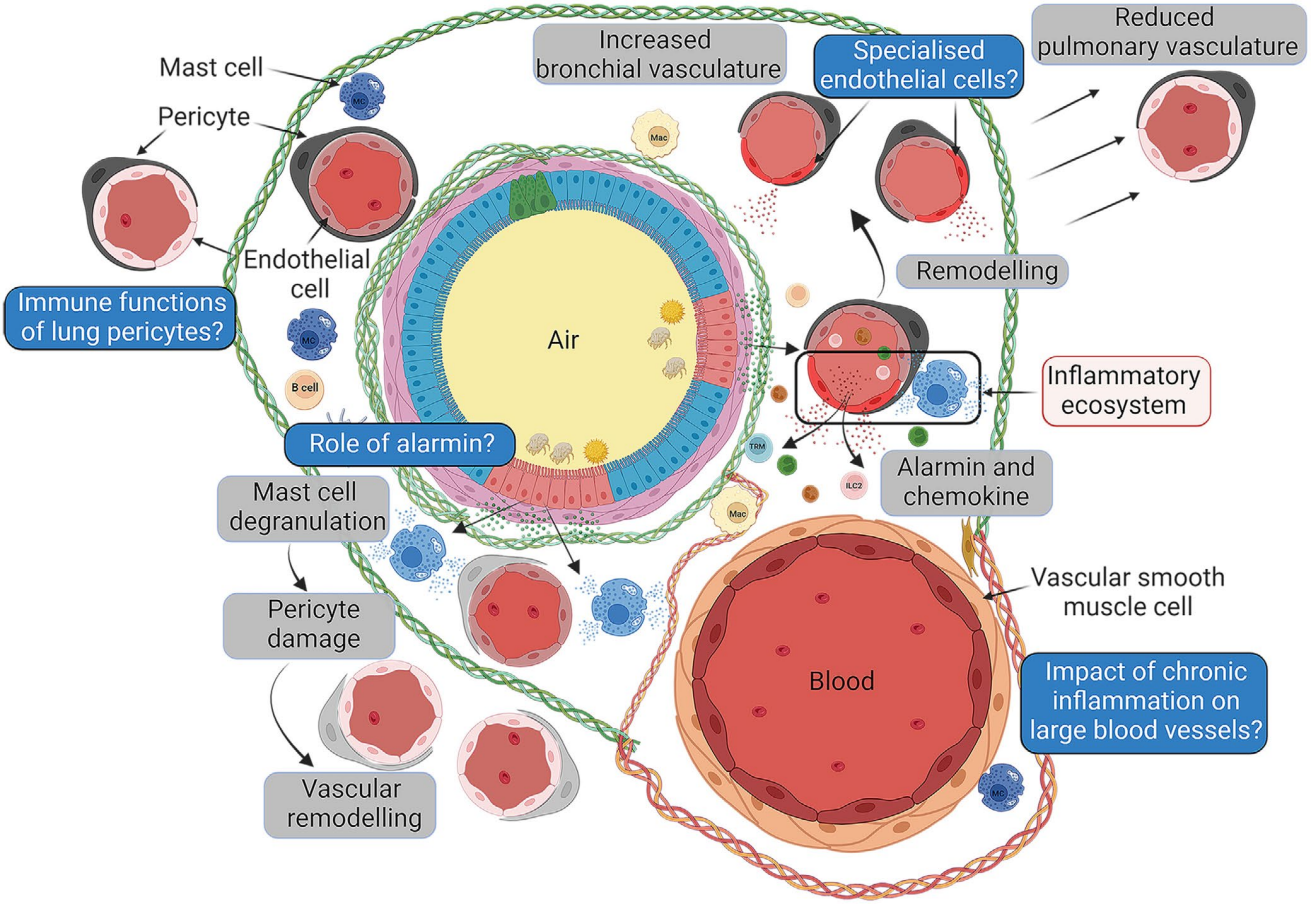
Complex role of the vasculature at the airway wall. Lung bronchi encompassing both bronchial and pulmonary vasculature impacted by asthma. Mural cells interact with local immune cells such as mast cells that together play a crucial role in the vascular remodeling process.

The lower airways are one of the most vascularized areas of all organs. Therefore, studying the pulmonary vasculature poses a challenge due to its extreme complexity and composition of all types of blood vessels, from capillaries to large arteries and veins. One approach to better understand the vasculature network at the airway wall is to discriminate between the presence of blood vessels from the bronchial and pulmonary circulation (Figure 4). These two circulations coexist in very close proximity but serve completely different purposes. Bronchial blood vessels supply large airways with nutrients and oxygen, whereas pulmonary circulation is responsible for gas exchange [79]. The presence of these different types and, in some cases, lack of distinction of these vessels has led to some confusion regarding whether an increase or decrease in vascularization is observed in asthma versus healthy lungs. A better spatial and temporal map of the lung vasculature in the context of chronic lung diseases is required to fully understand better this fundamental lung function.

### 5.1 Areas of Vascular Remodeling

All types of blood vessels are formed by a layer of endothelial cells delimiting the frontier between circulating blood and interstitial tissue. Endothelial cells are not a uniform population and have unique abilities to adapt to the type of blood vessels they are associated with (i.e., arterial, venular, lymphatic, or capillary). Recent scRNAseq studies have largely highlighted this heterogeneity both in human and mouse models. It is beyond the scope of this review to discuss individual lung endothelial cell phenotypes, and we refer to excellent works recently published [80, 81, 82, 83].

Increased vascularization is often noted as a key component of the extensive remodeling of the entire airway wall in asthma alongside changes to the epithelia, increased smooth muscle mass, and ECM geometry and deposition. This has been shown both in pediatric and adult asthma [84, 85, 86, 87]. One caveat of most of these studies is the type and diameter of blood vessels analyzed at the bronchial wall. While an increased number of capillaries will bring additional nutrients to facilitate airway remodeling, an increased number of larger postcapillary venules allows for the influx of infiltrating immune cells [88]. Historical data characterizing the pulmonary vasculature in lung disease have been dependent on data generated from thin tissue sections of human biopsies. To accurately map the true extent of the pulmonary vascular network, whereby the blood vessels wrap around the epithelial airways, 3D quantification is essential. Similarly, accurate detection of mural cells, such as pericytes and vascular smooth muscle cells, and specific markers for endothelial cell subtypes is fundamental to fully appreciating the vascular changes in disease.

Airway remodeling is a dynamic process and thus it is challenging to study in patient samples that generally only allow for a snapshot in time. During the progression of tissue remodeling, the active process of reforming and altering the tissue milieu to accommodate cell infiltration and proliferation, as well as matrix deposition, would “take the space” of existing lung tissue where most likely pulmonary vasculature was present. This would mean that at early time points during disease progression, mechanisms would have been initiated to remove existing blood vessels to accommodate the expansion of other cell types. Overall, this might indicate that the larger airways are characterized by a fine balance between bronchial and pulmonary circulation disruption during asthma progression. Exactly what the mechanisms are that underly this disturbance remains unclear, but potential targets are starting to be explored.

Using mouse models of allergic airway disease, precision cut lung slices (PCLS), and advanced imaging analysis approaches, we have analyzed changes to the vasculature in the context of early‐life allergen exposure. PCLS is an ideal tool we and others use to analyze the spatial distribution and interactions of stromal and immune cells, as robust and quantitative measurements are possible [30, 31, 89, 90]. We adapted PCLS to analyze the lung vasculature specifically around the large airways and adventitial cuff at the junctions between the bronchial and pulmonary vasculature. We showed that repeated exposure to HDM during the first few weeks of life led to a disruption of the pulmonary blood vessel network, which was characterized by a strong impact on the pericytes surrounding endothelial cells (discussed below). Discriminating the boundaries between bronchial and pulmonary circulation in mice is challenging as these structures are very close to each other. Improved whole‐organ imaging using light sheet microscopy will provide better mapping of the airway vasculature in the future.

Mast cells have been associated with blood vessel function for a long time, but the importance of these cells in driving changes in vascular structure is poorly understood [91, 92]. Mechanistically, mast cell proteases are known to be responsible for the loss of pericyte protrusions [32]. Mast cells have a unique repertoire of proteases that contribute to host defense against pathogens, and hypersecretion of these agents during asthma has been shown to exert a detrimental impact on the airway wall [93, 94]. Mast cell numbers are relatively low in mice, shown to predominantly align along the large airways, and are relatively scarce in the parenchyma. This is likely due to animals being housed in pathogen‐free facilities, leading to a less diverse microbiome. Data from mice have led to the conclusion that mast cells are rare in the lung [95], but this underestimates their abundance in human lungs. Indeed, our spatial transcriptomics data indicate a widespread abundance of mast cells in human samples throughout the lung, observed at both the large airways and alveolar areas [32].

Fundamentally, we have begun to reveal the molecular mechanisms regarding how chronic inflammation drives vascular remodeling in the context of early life, a critical period for lung development, immune cell maturation, and asthma pathophysiology. In this context, it is interesting to explore why lungs trigger the molecular mechanisms that initiate remodeling by an inflammatory response. It could be that this response is a form of protection against recurrent threats by creating a “buffer zone” and preventing access to pathogens to the more permeable pulmonary circulation deeper in the tissue, but this remains to be investigated.

The bronchial space in humans is more complex and contains different types of blood vessels compared to mouse large airways. Our spatial transcriptomics data surprised us in terms of the abundance of a vascular signal in human endobronchial biopsies [32]. Using cellular deconvolution and the signature of endothelial cells from published studies [96], we found endothelial cells in every region of interest analyzed. The presence of specialized endothelial cells within one type of blood vessel may drive areas of immune “hot spots” with increased immune cell recruitment in association with local areas of inflammation generated by basal cells and, as such, create spatial inflammatory ecosystems driving localized immune responses. Further spatial transcriptomics experiments, such as single‐cell spatial transcriptomics, will provide better identification of these cells and their cellular partners in health and asthma.

### 5.2 Role of Mural Cells at the Bronchial Wall

Mural cells are specialized cells present in close apposition to endothelial cells around all types of blood vessels. Two main cell types are characterized in this family: vascular smooth muscle cells present around large blood vessels such as arterioles, arteries, and veins, and pericytes surrounding precapillary arterioles, capillaries, and postcapillary venules [97]. They play a key role in vascular homeostasis and ensure the viability of endothelial cells by secreting proangiogenic factors and maintaining the architecture of blood vessels. The functions and roles of these cells have been extensively studied in highly vascularized organs such as the heart and brain. In the brain, they form the impermeable blood–brain barrier, and loss of pericyte functions in this organ emerges as one of the strongest markers for Alzheimer disease and vascular dementia [98, 99, 100, 101].

Pericytes have a highly coordinated spatial organization around blood vessels, and imaging is an essential tool for understanding their functions. For example, different morphological structures have been described for pericytes according to their localization: a mesh form in precapillary arterioles, thin strands in capillaries, and mesh in post‐capillary venules [102]. No specific markers have been defined for these cells, and localization is still the more reliable tool to identify them and analyze their functions. Emerging research will likely establish that these cells are highly heterogeneous transcriptionally, and further analysis will better define their molecular phenotypes and functions in health and disease. Most of our knowledge of mural cells comes from the field of neuroscience, yet vascular smooth muscle cells and pericytes have been implicated in the immune responses in multiple organs such as skin [103, 104] and muscle [105, 106].

Lung pericytes are enigmatic, despite being present in high abundance [107]. We know little about their abilities to regulate the organization of blood vessels at the airway wall and their response to inflammation during asthma [108]. In a mouse model of chronic exposure to HDM, blocking the Platelet‐derived growth factor receptor β (PDGFRβ) signaling pathway (main pericyte survival receptor) increased airway reactivity, suggesting that destabilizing pericyte function leads to worsening lung function [109].

Our recent work has focused on lung pericytes, and our data showed that they are present in high numbers in areas adjacent to the airway wall using PDGFRβ as a marker for localization. Interestingly, pericyte protrusions or the volume of cellular projections surrounding endothelial cells covered up to 60% of the surface of endothelial cells next to the large airways and 40% in the lung parenchyma. This suggests that pericytes in the lung adapt their protrusions in response to changes in their local environment, reminiscent of observations at the brain barrier. This shielding of endothelial cells by pericyte protrusions in the lung could represent a protective response due to the immediate exposure of the airways to the external environment and its associated threats.

Analyzing the functions of human lung pericytes remains a challenge since currently there are no specific or reliable human markers for pericytes commercially available. It is possible to obtain primary cells commercially, but with no definitive markers to assign identity and distinguish them from other mural cells or vasculature cells, conclusions drawn from experiments conducted with these cells are limited. Pericytes are highly abundant in mouse and human tissue; however, despite this, they do not appear as frequently as expected in publicly available scRNAseq studies. This could be due to either their relatively low mRNA content or poor viability following fluidic cell isolation. Nondissociative approaches such as spatial transcriptomics and multiplex proteomics may well shed new light on the importance of these cells in the lungs.

## 6 Perspectives and Unanswered Questions

Our knowledge of the structure and function of the airway wall has dramatically progressed over the past decades, leading to more precise and effective therapeutic interventions for patients with chronic lung diseases. The next generation of discoveries will be achieved by integrating data from more complex systems, and we believe spatial and temporal information will be key for future therapies. This will be achieved by improving the accessibility of research material and datasets to larger audiences involving both basic scientists and clinicians working on asthma. Hundreds of clinical trials have been performed for asthma, representing a unique resource to explore original elements of human lung biology and asthma using new technologies. We have described here recent discoveries relating to epithelial cells, ECM, and vasculature, but many more elements of the airway walls likely play critical roles in this microenvironment, such as nerve–immune interaction or the ever‐expanding role for fibroblasts, given their recent in‐depth subclassification [110]. Inhibiting inflammation has been achieved with great success in asthma, and our next challenge will be to understand and resolve the disrupted spatial organization of the airway wall to effectively treat the whole range of asthma endotypes.

The major unanswered questions related to spatial organization in asthma are as follows:

- Why are some airways more susceptible to remodeling and inflammation?
- How to detect these inflamed airways, ideally noninvasively, and is it possible to treat them locally?
- What are the early mechanisms of airway remodeling and how could we prevent or reverse it?
- Which therapy or combination of therapies influences the spatial organization of cells and restores normal airway wall distribution and lung function?

## Conflicts of Interest

The authors declare no conflicts of interest.

## Data Availability

Data sharing is not applicable to this article as no datasets were generated or analyzed during the current study.
